# Supplementary figures and images for: Effects of monocropping soil on plant growth and rhizosphere microbial community structure of Salvia miltiorrhiza Bge
Source: PeerJ. 2025 Nov 28;13:e20379. doi: 10.7717/peerj.20379 (PMC12667696; doi:10.7717/peerj.20379)

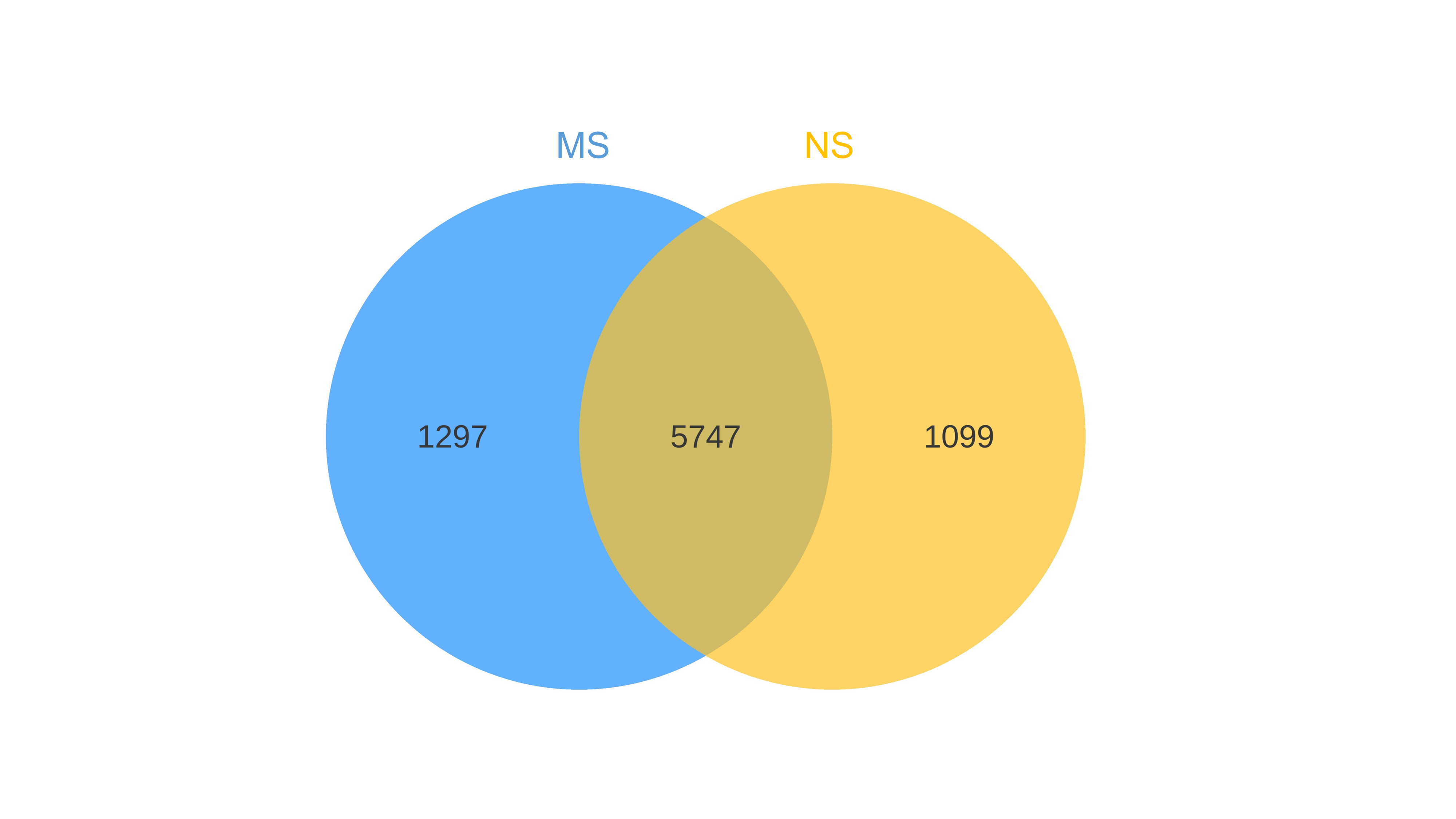

Supplement: Supplemental Information 1 [file peerj-13-20379-s001.png]

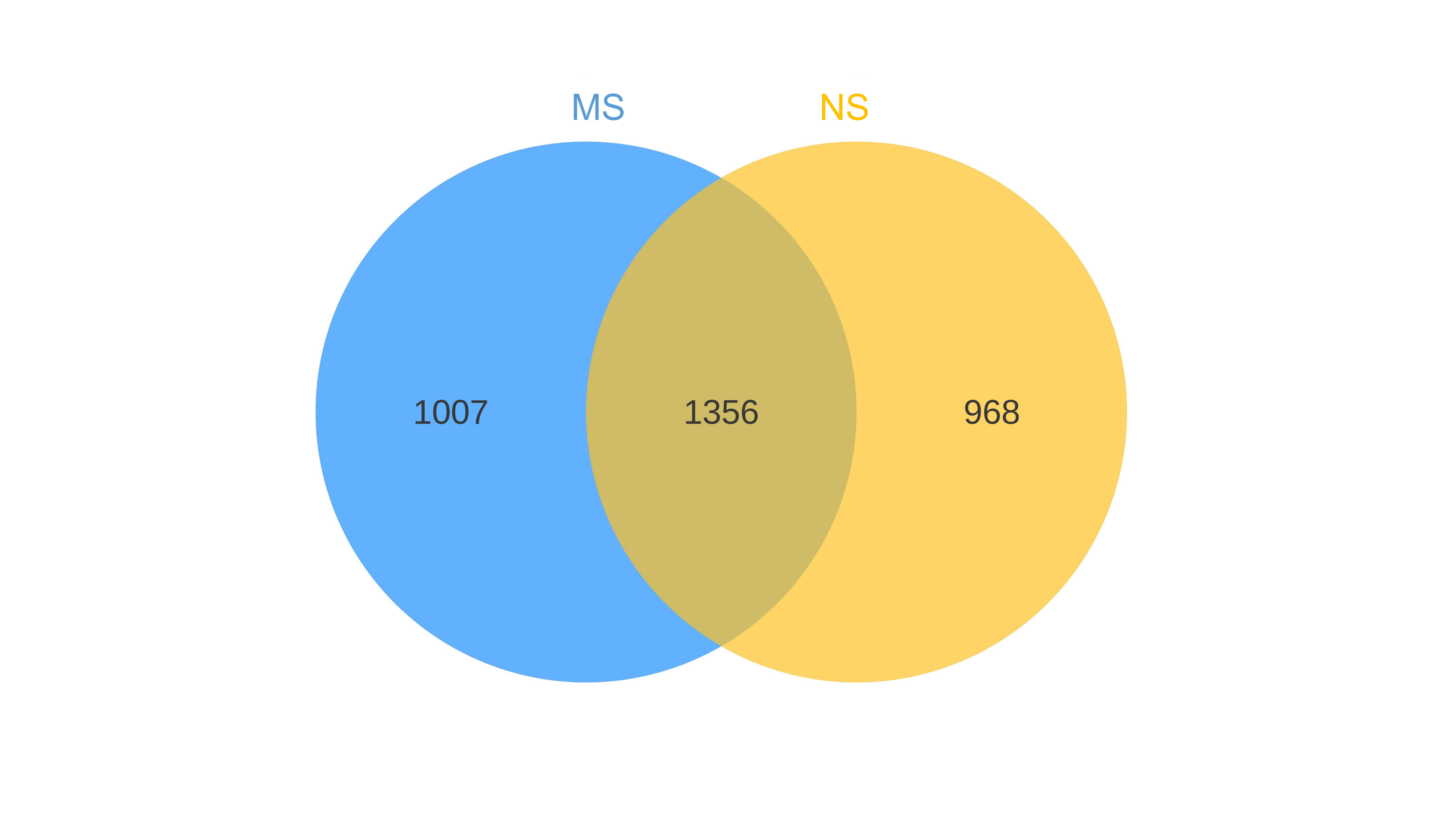

Supplement: Supplemental Information 2 [file peerj-13-20379-s002.png]
