# Supplementary material for: Effects of monocropping soil on plant growth and rhizosphere microbial community structure of Salvia miltiorrhiza Bge
Source: PeerJ. 2025 Nov 28;13:e20379. doi: 10.7717/peerj.20379 (PMC12667696; doi:10.7717/peerj.20379)
Supplement: Supplemental Information 3 — Note: Original tags = tag sequences obtained by splicing; effective label = the label sequence finally used for subsequent analysis after filtering the chimera; base = the base of the final valid data; effectiveness (%) = the percentage of the number of valid labels to the number of original PEs; oTUs = the number of operational taxonomic units. [file peerj-13-20379-s003.docx]

Table S1. Sequencing data statistics of bacteria and fungi in rhizosphere soil of continuous and non-continuous cropping of *Salvia miltiorrhiza*

| index | Bacteria | | Fungi | |
| --- | --- | --- | --- | --- |
|  | NS group | MS group | NS group | MS group |
| Raw Tags | 83256 | 82089 | 75428 | 74409 |
| Effective Tags | 77993 | 76756 | 74600 | 73511 |
| Base(nt) | 19741183 | 19424898 | 17312674 | 16595521 |
| Effective (%) | 92.52 | 92.32 | 89.00 | 86.85 |
| OTUs | 3942 | 4078 | 980 | 1017 |

Note: Original tags = tag sequences obtained by splicing; effective label = the label sequence finally used for subsequent analysis after filtering the chimera; base = the base of the final valid data; effectiveness (%) = the percentage of the number of valid labels to the number of original PEs; oTUs = the number of operational taxonomic units.
