# Supplementary material for: Effects of monocropping soil on plant growth and rhizosphere microbial community structure of Salvia miltiorrhiza Bge
Source: PeerJ. 2025 Nov 28;13:e20379. doi: 10.7717/peerj.20379 (PMC12667696; doi:10.7717/peerj.20379)
Supplement: Supplemental Information 4 [file peerj-13-20379-s004.docx]

Table S2 OTU statistics of bacterial and fungal communities in rhizosphere soil of continuous and non-monocropping of *S. miltiorrhiza*

| Microbial type | Treatment | Phylum | Class | Order | Family | Genus | Species |
| --- | --- | --- | --- | --- | --- | --- | --- |
| Bacteria | NS group | 66 | 150 | 307 | 420 | 644 | 288 |
|  | MS group | 70 | 154 | 306 | 413 | 628 | 278 |
| Fungi | NS group | 13 | 48 | 110 | 235 | 381 | 524 |
|  | MS group | 15 | 50 | 116 | 242 | 385 | 528 |
